# Examining the influence of team-based learning on medical students’ comprehension and attitudes regarding psychedelic therapies

**DOI:** 10.1080/19585969.2024.2398456

**Published:** 2024-09-02

**Authors:** Shiven Chaudhry, Anne E. Weisman, Molly Hagen, Kathryn L. S. Pauli, Burton J. Tabaac

**Affiliations:** a Benessair Health, Paradise Valley, AZ, USA; b Kirk Kerkorian School of Medicine, University of Nevada, Las Vegas, NV, USA; c Public Health, University of Nevada, Reno, Reno, NV, USA; d School of Medicine, University of Nevada, Reno, Reno, NV, USA; e Department of Neurology, Carson Tahoe Health, Carson City, NV, USA

**Keywords:** Medical Education, Psychedelics, Psychedelic-Assisted Therapy, team based learning

## Abstract

**Introduction:**

This study evaluates the impact of a two-hour team-based learning (TBL) curriculum on medical students’ knowledge, comprehension, ethical understanding, and attitudes towards psychedelic therapies.

**Methods:**

Sixty-three pre-surveys and fifty post-surveys assessed students’ perceived knowledge and attitudes using Likert scales. Forty-eight matched pre/post-knowledge tests with multiple-choice questions quantified changes in comprehension. The TBL approach featured independent learning, team readiness assessments, and application exercises.

**Results:**

Post-curriculum, students demonstrated significantly improved test scores (mean 41.4% increase, *p* < 0.0001) and more positive attitudes across 16 of 18 items (*p* ≤ 0.0495). Overall attitude scores increased 23% (*p* < 0.0001). Qualitative feedback reflected enhanced comfort discussing psychedelics clinically. While some students expressed support for psychedelic-assisted therapy, others cited reservations.

**Discussion:**

This innovative curriculum bridged an important education gap given the increasing relevance of psychedelic medicine. Findings suggest TBL enhances medical student preparedness in this emerging field. Continued curricular development is warranted to ensure proper psychedelic education aligns with patient needs and legislative policies. As psychedelic research progresses, maintaining instructional excellence is crucial for future healthcare professionals.

## Introduction

The term psychedelics refers to a broad category of psychoactive substances that can alter cognitive processes such as perception, mood, and emotions (D. E. Nichols 2004). While the use of psychedelics for spiritual exploration and healing in indigenous cultures dates back thousands of years, it wasn’t until the 1950s that researchers and clinicians developed an interest in their therapeutic potential (Doblin et al. 2019). However, after the initial surge of interest, psychedelic research experienced a significant slowdown due to growing concerns over safety profile, unregulated recreational use, government policies, and negative public opinion (Johnson et al. 2019).

More recently, attitudes towards psychedelics are shifting again. Since the early 1990s, there has been a notable resurgence of interest, commonly referred to the psychedelic renaissance, in utilising psychedelics as a therapeutic tool (Garcia-Romeu et al. 2016; Carhart-Harris and Goodwin 2017; D. Nichols et al. 2017; Nutt 2019; Nutt et al. 2020). Early data collected from recent preclinical and clinical trials suggest a therapeutic role of psychedelics in conditions including anxiety, depression, alcohol use, tobacco use, post-traumatic stress disorder (PTSD), and hospice care (Mithoefer et al. 2016; Ross et al. 2016; Luoma et al. 2020; Reiff et al. 2020; Davis et al. 2021; Maia et al. 2022; Holze et al. 2023). Along with increasing research, the public opinion and government policies are also evolving, making psychedelics more accessible to Americans. Use of psychedelics/hallucinogens for recreational use in young adults has increased dramatically over the last several years. Richmond (2021) notes 10% annual hallucinogen use in 2020 among young adults, reaching its peak since 1982. With an increasing number of cities now voting for decriminalisation and legislative changes, it is anticipated that the use of psychedelics will likely continue to rise in the future.

In light of this growing interest in regulated and unregulated use of psychedelics, there exists a significant gap in physician training on the science and medicine of psychedelic substances. While there are several private organisations that offer psychedelic training at a substantial cost, most medical schools do not offer any formal training as a standard part of the curriculum (Burgess et al. 2020). This deficiency in education poses a distinctive challenge given that substantial number of physicians will inevitably encounter patients who have either already engaged in psychedelics use or may express interest in the use of these substances. The primary objective of this study is to address this education gap within the medical field by developing a psychedelic course that explores a unique evidence-based curriculum design.

*Research questions:*

1. Does the implementation of a team-based learning methodology improve the comprehension, knowledge, and ethical understanding of psychedelics among medical students.
2. Do medical student perceptions/attitudes change on the use of psychedelics after the implementation of a Team-Based Learning (TBL) curriculum.

Team-based learning, as an active learning pedagogical approach, has gained considerable attention in the realm of phase one or pre-clinical medical education. Initially introduced over twenty years ago as a response to the overwhelming volume of content in medical education, an increasing number of medical schools are now adopting TBL formats to improve learner outcomes (Burgess et al. 2020). This pedagogical approach aids students to master learning objectives through a sequential process involving individual learning, team work *via* small group discussions, and immediate facilitator-led feedback (Parmelee et al. 2012). TBL is divided into three phases: (1) preparation phase, where learners review advance materials defined by faculty, (2) readiness assurance phase, where learners demonstrate knowledge through individual and group readiness assurance tests (RATs), and (3) application or individual summative testing phase, where participants apply concepts learned during the course (Burgess et al. 2020).

The present study introduces an innovative two-hour TBL curriculum at the Kirk Kerkorian School of Medicine (KKSOM) at the University of Nevada, Las Vegas (UNLV). The curriculum design was meticulously crafted with a conscious effort, recognising the need for sensitivity and thoughtful presentation when integrating psychedelics into coursework. In that regard, the course was predominantly shaped by students doing their own evidence-informed research on the subject matter with limited faculty guidance. The overarching goal was to develop a course that is impartial and strictly rooted in evidence. The hypothesis was that this flipped classroom style would improve medical students’ understanding and knowledge of psychedelic substances. Additionally, we expected changes in attitudes towards the use of psychedelics based on the current literature and the ongoing psychedelic renaissance movement.

## Methods

**Figure 1.**
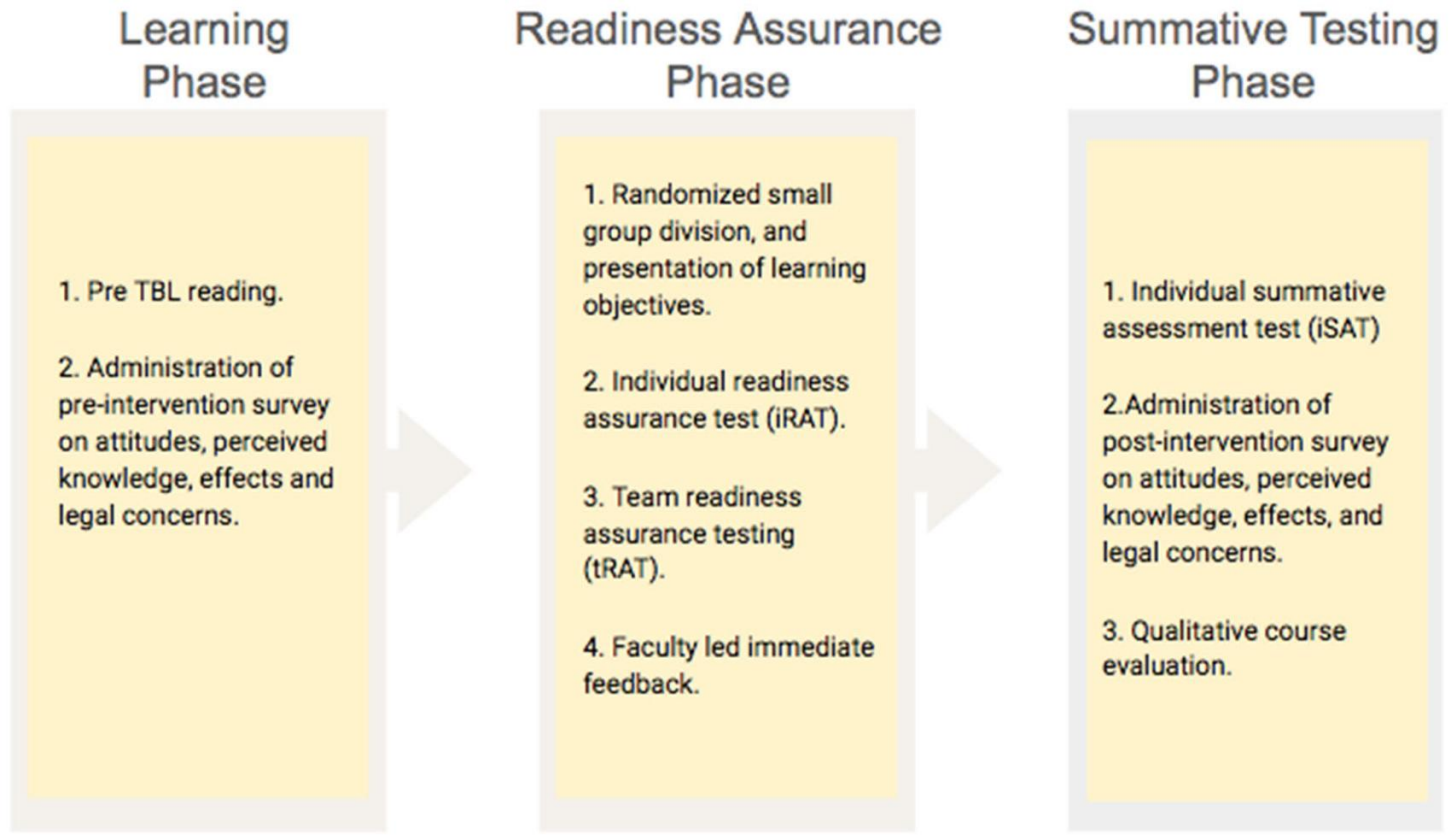
Schematic Outline of Team Based Learning

A multidisciplinary team designed and formulated this course to meet the educational needs of second year medical students at Kirk Kerkorian School of Medicine (KKSOM), University of Las Vegas (UNLV). As an integral part of the two-year integrative medicine curriculum, the course was incorporated into the psychiatry block to align with the overall education framework. The detailed learning objectives and course outline is presented in supplement information 1. The course was divided into three distinct phases: learning phase, readiness assurance phase, and summative phase, as outlined in Figure 1. A mixed methods research design was used. Prior to conducting the study, IRB was obtained for this survey study (UNLV-2022-293) at UNLV. As illustrated in Figure 1, surveys were distributed and collected at different time intervals to get pre and post-learning data. To ensure participant anonymity, surveys were collected in a de-identified manner using qualtrics software.

### Learning phase

In the initial learning phase, medical students received electronic access to reading material focused on psychedelic medicine and science. In addition to the required reading material, a survey on attitudes was administered, utilising a 5-point Likert scale ranging from ‘completely disagree’ to ‘completely agree’. The survey was adopted after a comprehensive literature review, building upon previous research conducted on cannabis and psychedelic attitude survey studies (Creswell and Creswell 2013; Barnett et al. 2018; Page et al. 2021; Barnett et al. 2022; Davis et al. 2022; Hearn et al. 2022; Li et al. 2023; Geller et al. 2024; Song-Smith et al. 2024). The survey, detailed in supplemental information 2, specifically examines attitude and self-assessment related to knowledge, comprehension, and ethical and legal considerations concerning the use of psychedelics.

### Readiness assurance phase

During the readiness assurance phase, second-year medical students were randomly divided into small groups of approximately 7 to 8 students each. A designated faculty introduced the learning objectives and course outline before presenting the study material. To gauge their baseline knowledge on psychedelics, each student took a 10-question multiple-choice quiz, which was then graded by the faculty.

The active learning component of the course followed the typical TBL structure. In the first portion of the TBL learning, each student took an individual reassurance test (iRAT) consisting of a fictitious clinical vignette followed by a series of questions (found in supplemental information 3). Students were permitted to use laptops and granted internet access to ensure evidence-based research. Each group then answered the same questions from the clinical vignette as a team i.e., team-based reassurance testing (tRAT) phase. Written responses were collected, and subsequently students engaged in faculty-led immediate feedback, involving an open question and answer format.

### Summative testing phase

In the summative testing phase, each student took a 10-question multiple-choice quiz on psychedelic knowledge, serving as a post-course knowledge assessment i.e., individual summative assessment test (iSAT). The initial subjective survey on attitudes was then administered to establish post-course attitudes. Lastly, students filled a survey providing course feedback which included qualitative commentary of the effectiveness of the course.

### Statistical methods

This study had two main inferential goals: to compare changes in student attitudes and knowledge regarding the use of psychedelics in medicine from before to after the training. Information for both outcomes was collected using a pre- and post-survey design. The pre-survey on attitudes also collected demographic data on participants including gender, age, race, and ethnicity (Table 1).

**Table 1.** Participant demographic data collected at baseline/pre-survey.

	All (*N* = 60)		Linked (*N* = 46)		Chi-squared
Characteristic	N	%	N	%	*p-*value
Gender					
Male	23	37.7	20	43.5	0.7738
Female	35	58.3	25	54.3	
Other^a,b^	2	3.2	1	2.2	
Race					
Asian	17	28.3	12	26.1	0.9579
Black^c^	1	1.7	0	0	
White	31	51.7	25	54.3	
Other	9	15.0	7	15.2	
Prefer not to answer^c^	2	3.3	2	4.3	
Ethnicity					
Hispanic	11	18.3	9	19.6	0.9999
Non-Hispanic	46	76.7	35	76.1	
Prefer not to answer^b^	3	5.0	2	4.3	
Age					
18–24	25	41.7	22	47.8	0.6231
25–34	33	55.0	22	47.8	
35–44^b^	1	1.7	1	2.2	
Prefer not to answer^b^	1	1.7	1	2.2	

^a^Other prefer not to answer (*n* = 2) and non-binary/third gender (*n* = 1).

^b^Not included in chi-square test due to low number of responses in category/level.

^c^Response(s) pooled with ‘Other’ for chi-square test due to low number of responses in category/level.

We quantified changes in attitudes using 18 survey items (Table 2). The attitude questions were asked using a five-point Likert scale which we coded to numerical equivalents such that higher scores indicate more positive attitudes about the use of psychedelics in medicine; items 1-4 and 10-18 were coded as ‘Strongly agree’ = 5, ‘Neutral’ = 3, and ‘Strongly disagree’ = 1 while items 5-9 were coded as ‘Strongly disagree’ = 5, ‘Neutral’ = 3, and ‘Strongly agree’ = 1. We then used numerical equivalents to calculate the average score for each question and overall for the pre and post-surveys. We linked pre and post-surveys for individuals using a unique identifier and calculated change by subtracting post-scores from pre-scores. Because our data were not normally distributed, we tested pre/post changes in student attitudes using Wilcoxon signed-rank tests.

**Table 2.** Summary statistics for numerical equivalents of the five-point Likert-scale survey questions with results of Wilcoxon signed-rank tests for pre/post training differences (*N* = 46 participants).

	Pre-survey		Post-survey		
Question	Mean	SD	Mean	SD	*p*-value
I am familiar with the possible therapeutic effects of psychedelics	3.15	1.30	4.15	0.79	**<0.0001**
I have substantial knowledge about psychedelics	2.09	1.24	3.63	0.93	**<0.0001**
I am extremely confident regarding my current knowledge of psychedelics	1.76	1.16	3.13	1.05	**<0.0001**
I have good knowledge of the side effects of psychedelics	2.35	1.29	3.83	0.85	**<0.0001**
Using psychedelics poses serious mental health risks	2.80	0.86	3.57	1.03	**<0.0001**
Using psychedelics poses serious physical health risks	2.89	0.92	3.37	1.04	**0.0040**
Psychedelics can be addictive	2.85	1.25	3.54	1.35	**0.0019**
I am concerned with psychedelics’ potential for abuse or misuse	2.52	1.17	3.13	1.24	**0.0019**
I am concerned about the potential side effects of psychedelic use	2.26	1.00	2.61	1.11	**0.0367**
Psychedelics have an acceptable role in medicine	3.41	1.13	4.04	1.15	**0.0004**
Psychedelics can help patients who suffer from chronic, debilitating medical conditions	3.87	0.93	4.20	0.93	0.0511
There are significant health benefits from using psychedelics	3.54	0.91	4.09	0.96	**0.0008**
Training about psychedelics should be incorporated in medical/health/social wellbeing related academic (preclinical) curricula	3.93	1.04	4.28	1.03	**0.0495**
Training about psychedelics should be incorporated into residency/field practice (clinical) requirements	4.04	0.89	4.30	1.05	0.0794
Psychedelics should be reclassified so that they are no longer schedule 1 drugs	3.39	1.14	4.20	1.00	**<0.0001**
Physicians should be able to legally prescribe psychedelics as medical therapy	3.46	0.81	4.13	0.86	**<0.0001**
Physicians should recommend psychedelics as medical therapy	3.39	0.77	3.91	0.91	**0.0007**
As a physician (in the future), I would be willing to help patients access psychedelics assisted therapy	3.67	0.97	4.11	0.88	**0.0006**
Overall *(summed score of all questions)*	55.4	12.3	68.2	12.5	**<0.0001**

*Note:* The bold numbers denote questions for which p value is less than or equal to 0.05.

We quantified changes in knowledge using a 12-item survey (Table 3). The knowledge questions were asked using various question formats, but in all cases, responses were coded as either correct or incorrect. Correct answers were assigned a ‘1’ and incorrect answers were assigned a ‘0’. We tested changes in correct versus incorrect answers on the pre and post-tests using McNemar exact tests for paired data. Following the tests for each knowledge question, we summed the number of correct answers on the pre and post-test and compared overall pre-/post-training differences using a Wilcoxon signed-rank test.

**Table 3.** Summary statistics for knowledge test questions answered correctly with results of McNemar exact tests for pre/post training differences (*N* = 48 participants).

	Pre-test		Post-test		
Question	N	%	N	%	*p*-value
1	3	6.3	7	14.6	0.2888
2	4	8.3	26	54.2	**<0.0001**
3	5	10.4	23	47.9	**0.0001**
4	28	58.3	33	68.8	0.3320
5	25	52.1	45	93.8	**<0.0001**
6	22	45.8	34	70.8	**0.0139**
7	17	35.4	22	45.8	0.2673
8	41	85.4	44	91.7	0.5050
9	40	83.3	46	95.8	0.1138
10	31	64.6	38	79.2	0.0961
11	35	72.9	41	85.4	0.1138
12	24	50.0	30	62.5	0.1814
Overall (*mean % correct, SD*)	5.73	1.66	8.10	1.93	**<0.0001^a^**

^a^Tested using Wilcoxon signed-rank test.

### Qualitative methods

To better understand the participants’ feedback from the course, the qualitative data from the post-surveys were coded using phenomenology. Phenomenology is the study of lived experiences (Creswell and Creswell 2013). Data were de-identified and coded. Once the initial coding was completed, the researcher grouped the codes into themes. Following this analysis, the data were again coded using ChatGPT. The researcher compared their themes to the themes that ChatGPT suggested and there was agreement in the results.

## Results

A total of 63 pre and 50 post attitudes surveys were collected. Data cleaning showed that only one participant had any item non-response (all other submitted surveys were 100% complete), that three duplicate pre-surveys and two duplicate post-surveys needed to be removed, and that 46 of the remaining 60 pre-surveys had post-surveys with matching participant identifiers. We compared demographic characteristics on the 46 linkable surveys with those on the full dataset of 60 surveys and found that there were no significant differences (Table 1, in all cases *p* ≥ 0.6231) suggesting that the subset of 46 linkable surveys was representative of the full sample. We therefore proceeded with the linked analysis approach which is more precise than a two-sample approach.

Participants were mainly female (*n*  =  25, 54%), white (*n*  =  25, 54%) or Asian (*n* = 12, 26%), non-Hispanic/Latino/Spanish (*n* = 35, 76.1%), and between the ages of 18 and 24 (*n* = 44, 96%; Table 1). There was only one non-binary participant and no African American participants.

When we compared the mean change in attitude survey questions we found an increase (i.e., more positive attitudes following training) on all 18-items, with 16 of the tests showing a significant positive change (in all cases *p* ≤ 0.0495). The two questions that did not show significant positive change had p-values of 0.0511 and 0.0794 corresponding to a raw increase of 8.5% and 6.4%. When we summed the numerical equivalents to each attitude question, an overall test showed highly significant positive change (*p* < 0.0001) and an average increase in overall scores of 23% (Table 2).

A total of 52 pre and 52 post knowledge surveys were collected. After one duplicate pre-test and three duplicate post tests were removed, the final sample was 48 surveys with no item non-response. Similar to the attitude survey, the percentage of participants who answered knowledge questions correctly increased on the post-surveys for all 12 questions. However, the effect size of the knowledge test was smaller for each individual question with only four of the 12 questions (33%) showing statistically significant change (in all other cases *p* ≥ 0.0961). The overall average number of correct questions on the post-survey was 41.4% higher than on the pre-survey, increasing from an average of 5.73 ± 1.66 SD to 8.10 ± 1.93 SD (*p* < 0.0001; Table 3).

Qualitative data from the post-surveys were analysed using phenomenology. Participants defined psychedelics and how they work with themes ranging from perception alteration, serotonergic effects, hallucinogenic properties, mechanisms of action, neuroplasticity and neurochemical effects, cognitive effects, and variability of effects. Diverse perspectives and levels of awareness regarding the legal and cultural aspects of psychedelic use in religious and ceremonial settings, include legal status and religious exemptions, Native American and Indigenous practices, legal precedents and court cases, variability in legal status, awareness of legal frameworks, along with beliefs and perceptions appeared in the data. Participants were able to identify different types of psychedelics, with responses reflecting the following themes: tryptamines, phenethylamines, lysergamides, dissociatives and others. There was a clear understanding of schedule one drug classification. The responses reflected awareness of the range of experiences following ingestion including hallucination, changes in perception, thoughts, emotions and cognition, euphoria, relaxation, heightened emotional states, detached feelings, distortions in time, changes in senses, increased heart rate, dilated pupils, changes in vitals and a range of adverse effects.

When provided with a clinical example and asked if they would enrol the patient in a clinical trial for psilocybin and to describe the mechanism of action along with the pharmacokinetics, participants were mostly in favour of the participant enrolling in the clinical trial. Participants cited potential for improvement in the patient’s depression and some included cautious optimism while emphasising the need for thorough screening and awareness based on the patient’s family history. Autonomy was named, citing the patient’s ability to make her own decisions once she was made aware of the potential risks. Some of the responses were against enrolment, citing concerns about the patient’s family history of schizophrenia, emphasising the need for careful screening and consideration of alternative methods. Others expressed a general hesitancy based on the lack of research and uncertainties given the family history. The remaining responses were neutral or negative, citing a need for more information or clearly stating that they were not confident making the clinical decision at this time.

In summary, while some medical students in this study express support or cautious optimism regarding enrolment in the clinical trial for psilocybin, others advise against it due to concerns about the patient’s family history and the current state of research. The participants’ responses from this class session reflected positive reception and enjoyment, engagement with the guest lecturer, with one participant stating, ‘It was great to run through cases and have someone with a high level of knowledge speak to us.’ The participants reflected their satisfaction with the educational value and learning experiences, with one participant stating, ‘Incredibly informative and fascinating.’ They also expressed a desire for more detailed content and structure and enjoyed the interactive and engaging format. One participant stated, ‘This definitely made me more comfortable discussing psychedelics with patients,’ and another stated, ‘I feel like it is educational and it is a topic that is applicable to the future as medicine continues to change’.

## Discussion

The pressing need to integrate psychedelic education into medical training is driven by the burgeoning interest in psychedelics in the United States. Medical students, both in the USA and abroad, are woefully unprepared for advances in psychedelic medicine, with previously published survey studies underscoring the paucity of basic science foundational knowledge (Geller et al. 2024; Song-Smith et al. 2024). The indicated interest to expand educational efforts and acknowledgment of the lacking framework to prepare clinicians as it pertains to psychedelics even expands into the postgraduate setting, with psychiatrists reporting their diminished insight and minimal awareness of the potential of psychedelic therapeutics (Barnett et al. 2018; Page et al. 2021; Barnett et al. 2022). Similar studies have surveyed psychologists in this regard (Davis et al. 2022; Hearn et al. 2022). The primary objective of this paper was to develop a comprehensive curriculum that responds to the dynamic needs of the community. With the recent breakthrough designations for MDMA and psilocybin in treating major depressive disorder and PTSD, respectively, we anticipate an increasing number of physicians encountering patients interested in psychedelic-assisted therapy. To address this, a primer geared towards medical professionals has recently been published to provide comprehensive reviews elucidating the unique properties and potential therapeutic applications of LSD, N,N-dimethyltryptamine (DMT) and ayahuasca, psilocybin, ibogaine, MDMA, and ketamine in an effort to better expand education and awareness (Beutler et al. 2024; Cherian et al. 2024; Evans et al. 2024; Muir et al. 2024; Shinozuka et al. 2024a, 2024b; Tabaac et al. 2024a, 2024b).

To meet these evolving needs, it is essential to incorporate psychedelic coursework as a standard component of medical education, ensuring an unbiased and conscientious approach. Our study focused on assessing the impact of a two-hour Team-Based Learning (TBL) course at the Kirk Kerkorian School of Medicine (KKSOM) on medical students’ knowledge, comprehension, and ethical understanding of psychedelic substances. The curriculum, formulated by a multidisciplinary academic faculty, aligned with the standards of phase one medical education. Comparing pre and post-testing results, we observed a significant improvement in test scores, indicating enhanced understanding and proficiency among students. This finding echoes previously published sentiment in assessing medical student and pharmacy student knowledge foundation (Li et al. 2023; Wang et al. 2023; Geller et al. 2024; Song-Smith et al. 2024). Subjective improvements were noted through survey studies, reflecting students’ positive perceptions of their knowledge post-course. Despite the time constraints, the curriculum strategically integrated basic learning on mood disorders, ensuring alignment with Liaison Committee on Medical Education (LCME) accreditation standards and including key concepts of physiology and pathophysiology, biochemistry, pharmacology, genetics, and integrative whole person care.

The TBL methodology in the two-hour format empowered students to navigate the coursework independently, utilising current data and literature. The course design, structured as clinical vignettes, facilitated a flipped classroom teaching approach, encouraging role-playing, evidence-based medicine application, problem-based learning, active discussions, and motivational interviewing. This innovative approach allowed for a more engaging and interactive learning experience, fostering a deeper understanding of the subject matter.

Qualitative data obtained through student feedback mechanisms revealed an overwhelmingly positive response, with students expressing engagement and relevance to their overall training. While some feedback highlighted timing concerns, we hope to address these in future course iterations. This work underscores that education regarding psychedelics is foundational and a crucial component to a curriculum of future clinicians given the persistent lack of awareness and judgements amongst healthcare providers and licenced professionals (Niles et al. 2021; Reynolds et al. 2021, 2022; Corrigan et al. 2022; Bhuiya et al. 2023).

As a secondary endpoint, we explored changes in attitudes and perceptions following the introduction of the psychedelic medicine course. While previous studies have examined general attitudes towards psychedelics, our study uniquely focused on the impact of proper education on attitude shifts. Encouragingly, our survey study indicated a positive shift in attitudes towards the therapeutic value of psychedelics, highlighting the potential long-term consequences on patient treatment and reducing bias towards psychedelic users. This builds on prior published work assessing attitudes and perceptions of a cohort in undergraduate college settings in regards to psychedelics and education as it pertains to attitudes (Wildberger et al. 2017).

Looking ahead, several critical questions remain for future exploration, including the optimal curricular time for psychedelic learning, the viability and repeatability of the course in other institutions, and the design of learning objectives aligned with evolving needs and legislative policies. Additionally, the long-term impact of this education on clinical practice and patient care should be assessed through longitudinal studies. We anticipate addressing these questions in our future studies, with a specific focus on safety and adverse events preparation, aiming to equip physicians with standardised education for the evolving landscape of psychedelic medicine. As the field of psychedelic medicine continues to advance, it is imperative that medical education keeps pace, ensuring that future healthcare professionals are well-prepared to navigate this exciting and potentially transformative area of medicine.
